# Transversus abdominal plane (TAP) block for postoperative pain management: a review

**DOI:** 10.12688/f1000research.7015.1

**Published:** 2015-11-26

**Authors:** Jan Jakobsson, Liselott Wickerts, Sune Forsberg, Gustaf Ledin

**Affiliations:** 1 Department of Anaesthesia & Intensive Care, Institution for Clinical Science, Karolinska Institute at Danderyds and Norrtälje Hospitals, Stockholm, Sweden

**Keywords:** TAP-block, Transversus abdominal-plane block, ultra-sound, pain managment

## Abstract

Transversus abdominal plane (TAP) block has a long history and there is currently extensive clinical experience around TAP blocks. The aim of this review is to provide a summary of the present evidence on the effects of TAP block and to provide suggestions for further studies.

There are several approaches to performing abdominal wall blocks, with the rapid implementation of ultrasound-guided technique facilitating a major difference in TAP block performance. During surgery, an abdominal wall block may also be applied by the surgeon from inside the abdominal cavity.

Today, there are more than 11 meta-analyses providing a compiled evidence base around the effects of TAP block. These analyses include different procedures, different techniques of TAP block administration and, importantly, they compare the TAP block with a variety of alternative analgesic regimes.

The effects of TAP block during laparoscopic cholecystectomy seem to be equivalent to local infiltration analgesia and also seem to be beneficial during laparoscopic colon resection. The effects of TAP are more pronounced when it is provided prior to surgery and these effects are local anaesthesia dose-dependent. TAP block seems an interesting alternative in patients with, for example, severe obesity where epidural or spinal anaesthesia/analgesia is technically difficult and/or poses a risk. There is an obvious need for further high-quality studies comparing TAP block prior to surgery with local infiltration analgesia, single-shot spinal analgesia, and epidural analgesia. These studies should be procedure-specific and the effects should be evaluated, both regarding short-term pain and analgesic requirement and also including the effects on postoperative nausea and vomiting, recovery of bowel function, ambulation, discharge, and protracted recovery outcomes (assessed by e.g., postoperative quality of recovery scale).

## Introduction

There is an increasing interest in abdominal wall block. The ultrasound-guided technique has improved the performance and success rate. The effects, advantages and potential disadvantages as compared to alternative pain management are, however, not obvious. There are several alternatives and the best technique is not clear. The ultrasound guidance has made this block more attractive. There are today several videos describing anatomy and how to perform the TAP block effectively ( https://www.youtube.com/watch?v=9TIHDn7uBZI, https://www.youtube.com/watch?v=CzMDdrPbLEM). Not introducing the needle too close to the probe is important in order to visualise the needle reaching the layer between the internal oblique and transverse abdominal muscle ( https://www.youtube.com/watch?v=6E3ynIn6Ud4).

Our aim in this paper is to present the benefits and merits of TAP block in adults. It will provide the readers a review of techniques and outcomes from available studies. There are more than 11 meta-analyses available compiling studies assessing TAP blocks. This paper will provide summaries of these meta-analyses and suggest best practice. It will also suggest areas where there is a need for further high-quality studies.

## What we know now: meta-analyses and systematic reviews

There are 11 published meta-analyses around the effects of TAP block. The most recent was published in September 2015 ^1^ and the first was a Cochrane review by Charlton *et al.* published in 2010 ^2^. They assess the effects of various abdominal blocks, most provided by ultrasound-guided technique. The studies are, however, hard to compare as different approaches, local anaesthetic concentrations, and volumes have been used. The abdominal block has been compared to “placebo” or to other anaesthetic techniques, for example, local infiltration analgesia or intrathecal anaesthesia. The most recent meta-analysis by Baeriswyl *et al.* was published in the September 2015 issue of Anaesthesia and Analgesia ^1^. It included 31 controlled trials and 611 adult patients in all. Its primary focus was on the opioid-sparing effects, and the cumulative morphine consumption at 6 hours postoperatively, and its secondary objectives were 24-hour morphine consumption, pain ratings, and postoperative nausea and vomiting. It showed that the ultrasound-guided TAP block was associated with a reduced IV morphine consumption at 6 hours postoperatively by a mean difference of 6 mg, independent of the type of surgical anaesthesia (general anaesthesia, spinal anaesthesia with or without intrathecal long-acting opioid). The beneficial effect of cumulative morphine consumption was also seen at 24 hours (mean 11 mg). Pain ratings were reduced at 6 hours postoperatively but no effect was seen in the incidence of postoperative nausea and/or pruritus, either at rest or during movement. The authors conclude, “Ultrasound-guided TAP block provides marginal postoperative analgesic efficacy after abdominal laparotomy or laparoscopy and cesarean delivery. However, it does not provide additional analgesic effect in patients who also received spinal anesthesia containing a long-acting opioid”. Thus the result of this most recent review is in line with the ones previously published.

Charlton *et al.* published a Cochrane systematic review in 2010 assessing the effect of TAP block for pain relief after abdominal surgery ^2^. They included 8 prospective randomised studies. A clear opioid-sparing effect was found as compared to placebo or “no block”. Compared with no TAP block or saline placebo, TAP block resulted in significantly less postoperative requirement for morphine at 24 hours (mean difference -21.95 mg) and 48 hours (cumulative difference -28.50 mg). No effect was found on nausea and vomiting. The authors requested further studies comparing TAP block with alternative local anaesthesia techniques, for example, local infiltration and single-shot intrathecal anaesthesia.

Siddiqui *et al.* published a second meta-analysis in 2011 around the efficacy of the TAP block ^3^. Four studies were included; laparoscopic cholecystectomy, caesarean section with Pfannenstiel incisions, total abdominal hysterectomy, and large bowel resection midline incision. It was found that patients that were randomised to active TAP block had a significantly lower cumulative morphine need during the first 24 hours post surgery ( *P*<0.001), a significantly longer time until they needed rescue morphine ( *P*<0.001), as well as less pain up to 24 hours post surgery. No significant effects from the TAP block were noticed in postoperative nausea and vomiting. The most profound TAP block effects were noticed for the caesarean section and colon surgery.

Mishriky *et al.* published a third meta-analysis in 2012, looking at analgesia after caesarean section. Nine studies were included ^4^. They found that TAP block significantly reduced opioid consumption (mg morphine equivalents) after caesarean section. The mean difference in opioid need was -10.18, -13.83, and -20.23 mg at 6, 12, and 24 hours respectively. TAP block also reduced pain during the first 12 hours and reduced nausea among the patients who did not have intrathecal morphine. The combination of TAP block and spinal morphine was associated with a small reduction in pain during movement in the first 6 hours after surgery. Intrathecal morphine was, however, more effective; it was associated with a lower pain score and opioid consumption at 24 hours after surgery. The intrathecal morphine group also had a longer time before the first rescue analgesia request. The intrathecal morphine caused more morphine-related side effects.

Johns *et al.* conducted a fourth meta-analysis published in 2012 looking at the analgesic effects of TAP block after abdominal surgery ^5^. In all, 9 studies representing both published and unpublished results were analysed including 413 patients; 205 that had a TAP block and 208 control patients. TAP block was found to be safe and effective and was associated with a significantly lower morphine need both 24 and 48 hours after surgery, -23.71 mg ( *P*<0.002) and -38.08 mg ( *P*<0.0001) respectively, and also a lower incidence of postoperative nausea and vomiting. Pain scores did not differ significantly.

Abdallah *et al.* published a meta-analysis in 2012 assessing TAP block for postoperative analgesia after caesarean delivery performed under spinal anaesthesia ^6^. They analysed the results for 5 studies including 312 patients. TAP block was found to reduce the mean first 24-hour post surgery cumulative morphine need by 24 mg when intrathecal morphine had not been used. TAP block also lowered pain scores (0.8/10) and morphine related adverse effects. The effects of TAP block were not significantly different from intrathecal morphine. It was concluded that TAP block can reduce morphine need during the first 24 hours after surgery when intrathecal morphine is not used.

Abdallah *et al.* published another meta-analysis in 2013 focusing on the difference between two TAP block approaches (the posterior and the lateral) and their effect on the duration of pain relief after lower abdominal surgery incision ^7^. In all, 12 randomised studies were included in the analysis (641 patients); 4 studies with a posterior TAP technique and 8 with a lateral technique. They found the posterior approach was associated with a significantly lower morphine need both 12 to 24 hours and 24 to 48 hours after surgery; a mean difference of 9.1 mg ( *P*<0.02) and 5 mg ( *P*<0.03), respectively. The posterior TAP block also had significant effects on pain, reducing pain scores at rest and during movement at 24, 36, and 48 hours after surgery. The lateral TAP was not associated with any significant differences.

De Oliviera *et al.* published a 7th meta-analysis assessing TAP block analgesic effects after laparoscopic surgery ^8^. They included 10 randomised studies covering 633 patients. They found TAP block to lower pain at rest -2.41/10 during the first 4 hours postoperatively and -1.33/10 at 24 hours post surgery. TAP also reduced IV morphine need (weighed mean -5.74 mg morphine equivalents). It was also found that TAP block administered preoperatively was more effective on pain, and reduced postoperative morphine consumption when compared with blocks placed postoperatively. No local anaesthesia toxicity was reported.

Zhao *et al.* published an 8th meta-analysis assessing TAP block for postoperative analgesia after laparoscopic surgery ^9^. In all, 14 studies with a total of 905 patients were included in this analysis. TAP block resulted in significantly less postoperative analgesic consumption at 24 hours (mean difference = -25.46, *P*<0.00001), and less patients requiring analgesic postoperatively ( *P*=0.03). TAP block reduced pain; pain scores were significantly different at 2 hours (mean difference = -1.55, *P*<0.00001). A borderline difference between the active TAP block and control was seen at 6 hours (mean difference = -1.13, *P*=0.05). TAP block had no effect on pain at 24 hours. TAP block was associated with significantly more postoperative nausea and vomiting (odds ratio 2.04, *P*=0.34). The authors concluded TAP reduced 24-hour analgesic requirements, had minor effects on early pain, and may increase the risk of postoperative nausea and vomiting.

Yu *et al.* published the 9th meta-analysis in December 2014 assessing TAP block as compared to local wound infiltration analgesia in patients undergoing lower abdominal surgery ^10^. In all, 4 randomised studies were included in this analysis; 96 patients having a TAP block and 100 patients having local infiltration analgesia. The TAP block reduced pain, pain scores were lower both at rest and during movement as compared to local infiltration analgesia at 24 hours postoperatively; weighed mean difference -0.67 ( *P*<0.01) and -0.89 ( *P*<0.01) respectively. Postoperative 24-hour morphine need, incidence of postoperative nausea and vomiting, and pain assessed by the visual analogue scale score at 2 and 4 hours did not differ between the TAP and local infiltration analgesia groups of patients.

Ripollés *et al.* published an update and summary of the existing evidence in early 2015 and indeed supports TAP block’s beneficial effects ^11^. The analysis was based on prospective randomised studies published between 2007 and 2013 in English or Spanish with a Jadad score of >1. Studies in adult patients including ultrasound-guided blocks compared to other analgesic techniques were assessed. In all, 28 randomised clinical trials were included in the analysis. There was a huge heterogeneity in study design. The studies used different TAP techniques, local anaesthetic concentrations as well as volumes, and also comparators differed. Most studies compared the TAP block against placebo but there were also studies comparing the TAP block against epidural analgesia, local infiltration, and ileoinguinal-ileohipogastric block. Outcomes studied were opioid consumption, and pain at rest or during movement. However, the results were not entirely congruent, although most studies did see some beneficial effects. These authors did, however, conclude that TAP block is an effective technique for reducing opioid use postoperatively following colorectal surgery, caesarean section, cholecystectomy, hysterectomy, appendectomy, donor nephrectomy, retropubic prostatectomy, and bariatric surgery. They did see obvious gaps: the data found in available randomised clinical trials was not considered fully conclusive. These authors suggest that there is a need to develop new and well-designed randomised clinical trials, with enough statistical power to compare different approaches, drugs, doses, and volumes for the same intervention, aiming to answer the current questions and assess the effect of TAP-block effects in routine clinical practice.

## Discussion

There has been an increasing interest in the transversal abdominal plane block during the last decade. It seems reasonable to conclude that TAP block is a safe technique; no significant side effects have been reported ( Table 1). The block provides an opioid-sparing effect, but the effects on opioid-related side effects, postoperative nausea and vomiting, and bowel function are not fully consistent. The effects on early postoperative pain and reduced opioid consumption 24–48 hours after surgery are seemingly similar to single-shot spinal anaesthetic with intrathecal morphine and more or less equal to local infiltration analgesia.

**Table 1.** Meta-analyses on TAP-block.

Publication	Year	Main objective	No. of studies	Conclusion
**^11^Analgesic efficacy of the ultrasound-guided blockade** **of the transversus abdominis plane – a systematic** **review** *Ripollés, Mezquita, Abad and Calvo* *Brazilian journal of anesthesiology 2015, **65**: 255–80*	2015	To determine the efficacy of the ultrasound-guided transversus abdominis plane (TAP) blockade for different surgical interventions, as well as the indications according to the approaches and their influences.	31 RCTs, including 2193 patients (28 RCTs with mid-axillary approach, 1 with subcostal, and 2 RCTs with oblique subcostal approach).	TAP block has been shown to be an effective technique in colorectal surgery, caesarean section, cholecystectomy, hysterectomy, appendectomy, donor nephrectomy, retropubic prostatectomy and bariatric surgery. However, the data found in randomized clinical trials are not conclusive.
**^10^Transversus abdominis-plane block versus local** **anaesthetic wound infiltration in low abdominal** **surgery: a systematic review and meta-analysis of** **randomized controlled trials** *Yu, Long, Lujan-Hernandez, Succar, Xin and Wang* *BMC Anaesthesiology 2014, **14**: 121*	2014	To compare the efficacy of single- shot TAP block with that of single- shot local anaesthetic infiltration for postoperative analgesia in adults.	4 RCTs, encompassing 96 TAP block and 101 local anaesthetic infiltration patients.	TAP block and local anaesthetic infiltration provide comparable short-term analgesia, however TAP block has better long-term effects.
**^9^Transversus abdominis plane block for postoperative** **analgesia after laparoscopic surgery: a systematic** **review and meta-analysis** *Zhao, Tong, Ren, Ding, Wang, Zong, Jin and Li* *Int. J Clin Exp Med 2014, **7**(9): 2966–2975*	2014	To determine the effect of the TAP block on postoperative opioid consumption and the number of patients requiring opioids after laparoscopic surgery.	14 trials with 905 patients.	TAP block, as a part of a multimodal analgesic regimen, results in less analgesic consumption, less requirement of analgesic, and less pain at 2 hours and slightly less at 6 hours but not after 24 hours following laparoscopic surgery, in comparison with usual care alone or placebo block. In addition TAP block can increase the incidence of postoperative nausea and vomiting.
**^8^Transversus abdominis plane block to ameliorate** **postoperative outcomes after laparoscopic surgery: a** **meta-analysis of randomized controlled trials** *De Oliveira, Gildasio, Castro-Alves, Nader, Kendall and* *McCarthy* *Anaesth. Analg 2014, **118**: 454–63*	2014	To evaluate the effect of TAP block on postoperative analgesia outcomes for laparoscopic surgical procedures.	10 RCTs with 633 subjects were included.	TAP block is an effective strategy to improve early and late pain at rest and to reduce opioid consumption after laparoscopic surgical procedures. Preoperative administration of a TAP block seems to result in greater effects on postoperative pain outcomes. Local anaesthetic showed a dose response effect on late pain and postoperative opioid consumption.
**^7^Duration of analgesic effectiveness after posterior** **and lateral abdominis plane block techniques for** **transverse lower abdominal incisions: a meta-analysis** *Abdallah, Laffey, Halpern and Brull* *BJA 2013, **111**: 721–35*	2013	To examine the duration of analgesia associated with posterior and lateral TAP blocks in the first 48 hours after lower abdominal transverse incision surgery.	12 RCTs, including 329 patients in the TAP group and 312 in the control group.	TAP block using the posterior approach reduced the rest and dynamic pain as well as the consumption of morphine for up to 48 hours. The effect was not seen when a TAP block was performed using the lateral approach.
**^6^Transversus abdominis plane block for postoperative** **analgesia after Cesarean delivery performed under** **spinal anaesthesia? A systematic review and meta-** **analysis** *Abdallah, Halpern and Margarido* *BJA 2012, **109**(5): 679–87*	2012	To examine whether TAP block can reduce IV morphine consumption in the first 24 hours after caesarean delivery.	5 trials including 312 patients.	TAP block reduced IV morphine consumption and pain scores in the first day after surgery. TAP block can provide effective analgesia after caesarean delivery when intrathecal morphine has not been used.
**^5^Clinical effectiveness of transversus abdominis plane** **(TAP) block in abdominal surgery: a systematic review** **and meta-analysis** *Johns, O´Neill, Ventham, Barron, Brady and Daniel* *Colorectal Dis. 2012, **14**(10): e635–42*	2012	To determine the effect of TAP block on morphine requirements 24 hours after abdominal surgery.	9 studies. 205 patients received a TAP block and 208 a placebo.	TAP block is safe, reduces postoperative morphine requirements, nausea and vomiting and possibly the severity of pain after abdominal surgery.
**^4^Transversus abdominis plane block for analgesia after** **cesarean delivery: a systematic review and meta-** **analysis** *Mishriky, MD, George, MD, Habib, MBBCh.* *Can J Anesth. 2012, **59**: 766–778*	2012	To assess the efficacy of TAP block in improving analgesia after caesarean delivery.	9 studies. 261 patients received TAP block and 263 served as controls.	TAP block significantly improved postoperative analgesia in women undergoing caesarean delivery who did not receive intrathecal morphine but showed no improvement in those who received intrathecal morphine. Intrathecal morphine was associated with improved analgesia compared with TAP block alone at the expense of an increased incidence of side effects.
**^3^A meta-analysis on the clinical effectiveness of** **transversus abdominis plane block** *Siddiqui, Sajid, Uncles, Cheek and Baig* *J Clin Anesth. 2011, **23**(1): 7–14*	2011	To study the efficacy of the TAP block.	4 studies. 86 patients in the TAP block group and 88 in the non-TAP block group.	Patients with TAP block required less morphine after 24 hours than those who did not have the block (random effects model: SMD[AU: Please define] -4.81, 95% confidence interval [-7.45, -2.17], z = -3.57, *P*<0.001). No statistical differences were found with respect to nausea.
**^2^Perioperative transversus abdominis plane (TAP)** **blocks for analgesia after abdominal surgery** *Charlton, Cyna, Middleton and Griffiths* *Cochrane Database Syst Rev. 2010, **8**(12): CD007705.* *DOI: 10.1002/14651858.CD007705.pub2*	2010	To assess the effects of TAP blocks (and variants) on postoperative analgesia requirements after abdominal surgery.	8 studies (358 participants), 5 assessing TAP blocks, 3 assessing rectus sheath blocks.	Compared with no TAP block or saline placebo, TAP block resulted in significantly less postoperative requirement for morphine at 24 hours (mean difference -21.95 mg, 95% confidence interval -37.91 to 5.96; 5 studies, 236 participants) and 48 hours (mean difference -28.50, 95% confidence interval -38.92 to -18.08; 1 study of 50 participants) but not at 2 hours (all random-effects analyses). As with TAP blocks, rectus sheath blocks made no apparent impact on nausea and vomiting or sedation scores.

There are obvious factors to consider before the implementation of TAP block in routine clinical practice; patient- and surgery-related factors, alternatives, and the technique to be used (see Table 2). The introduction of the ultrasound-guided block technique has made the TAP block an interesting option as part of multimodal postoperative pain management, partly because of its technical simplicity. The ultrasound technique has made the TAP block easier to perform but it is at present not possible to provide any firm data showing a higher efficacy for the ultrasound-guided techniques. Ultrasound-guided bilateral TAP block is commonly performed with a high-frequency linear ultrasound probe and an in-plane needle guidance technique ^12^. The TAP block provides effective analgesia with opioid-sparing effects. Disadvantages include the need for a bilateral block for midline incisions and the absence of effectiveness for visceral pain ^13^. The effect of the block is dependent on the technique used and patient anatomy. Støving *et al.* studied the effect of TAP block in healthy volunteers. They found huge inter-individual variability in objective sensory block and duration of effect ^14^. The TAP block is a volume block and is performed by injecting local anaesthetic solutions in the transverse abdominis plane without specific reference to the nerves responsible for the innervations of the abdomen wall. It is not surprising that in these conditions the quality of a TAP block is dependent on the approach and the volume administered. There are more specific approaches: blocks specifically blocking the involved innervation of the abdomen include ilioinguinal, iliohypogastric, and/or intercostal blocks that can be used alone and/or in combination depending on the surgical incision. Early reduced opioid consumption is a consistent finding associated with the TAP block. These effects are seemingly most pronounced in transverse incisions, when the block is provided prior to surgery, and there is also a dose effect. Kokulu *et al.* showed that TAP block provided preoperatively was associated to a reduction of desflurane need and thus was cost effective during elective cholecystectomy ^15^. It is somewhat surprising that the effects on postoperative nausea and vomiting are not entirely conclusive: one meta-analysis even suggested there was no benefit and another a potential increase in postoperative nausea and vomiting in the TAP groups.

**Table 2.** Considerations around TAP-block.

**Patient**	Lean *vs.* obese
	Unsuitable for spinal
**Surgery**	Upper *vs.* lower abdominal
	Open *vs.* minimally invasive
	Midline or lateral or Pfannenstiel incision
**Pain management/Comparator;** **alternative**	Local infiltration analgesia
	Paravertebral block
	Spinal
	Epidural
	PCA[AU: Please define] morphine
**Technique and drugs**	Ultrasound *vs.* blind
	Lateral *vs.* posterior
	Local anaesthetic • Concentration and volume • Adjuncts

The long-term effects require further studies. Keller *et al.* found, studying 200 consecutive patients who underwent a laparoscopic colorectal resection, that adding TAP blocks to an enhanced recovery pathway facilitated shorter length of hospital stay with lower readmission and reoperation rates, when compared to previously published series. This suggested TAP blocks might be an efficient, cost-effective method for improving laparoscopic colorectal surgery results ^16^. Similar positive experiences were reported by Favuzza *et al.* ^17^. Both these studies compared a TAP block performed at the end of surgery with the laparoscope still in place, actually visualising the muscle layers of the structures from the “inside”, thus not performed by ultrasound technique but by the surgeon. The surgeon performed the block from the outside, passing the needle through the skin, mid-axillary, approximately half the distance between the iliac crest and the costal margin. The traditional two pops technique, passing two fascia borders, was used and the injection of 30 ml of 2.5 mg/ml bupivacaine was injected under surveillance of the laparoscope, imaging the spread at the place for the transversus abdominis muscle. It should be acknowledged that these studies were not randomised or blinded.

The analgesic effect has a duration related to the local anaesthetic administered. The analgesic duration has been shown to be longer when dexamethasone is added to local anaesthesia ^18^. There are also positive results from the use of liposomal bupivacaine: the total opioid use in the first 72 hours after injection was significantly decreased in the group that received liposomal bupivacaine compared to non-liposomal bupivacaine. Patients in the liposomal bupivacaine group had significantly lower maximal pain scores at all time periods studied, as well as a decreased incidence of nausea/vomiting. There was a trend toward decreased length of stay in the liposomal bupivacaine group ^19^. The addition of sufentanil to bupivacaine did not provide longer or more effective effects as compared to bupivacaine alone for pain management following laparoscopic cholecystectomy ^20^.

Today, there are several meta-analyses around the effects of TAP block, but still, the exact place for TAP block requires further studies. The last meta-analysis by Ripollés *et al.* concludes that the data found in randomised clinical trials are not conclusive and, as a result, it is necessary to develop new and well-designed randomised clinical trials, with enough statistical power to compare different approaches, drugs, doses, and volumes for the same intervention, aiming to answer the current questions and monitoring their effects in routine clinical practice. The TAP block as part of a multimodal analgesic strategy, as compared to local wound infiltration analgesia, and spinal/intrathecal analgesia (IT morphine), not only improved morphine consumption during the first 24 to 48 hours but also quality of recovery assessed in a broader and more protracted/long-term time perspective, for example, assessed by postoperative quality of recovery scale ^21^. It seems it would also be of value to conduct studies comparing the TAP block to paravertebral block. Chelly *et al.* have shown that the paravertebral block was effective for pain management following open radical retropubic prostatectomy ^22^. There is also a need to better explore the place for TAP block in paediatric perioperative care ^23^.

## Conclusion

In conclusion, TAP block is a safe and interesting block that may be provided by ultrasound-guided technique or intraoperatively by the surgeon, providing postoperative analgesia, and a reduced need for morphine analgesia during the first 24 to 48 hours following abdominal procedures. TAP block administered prior to surgery reduces not only postoperative opioid requirements but also intraoperative anaesthetic needs. There is, however, a need for further high-quality studies assessing the effects of TAP block as part of multimodal analgesia, and as compared to local infiltration analgesia and intrathecal morphine, assessed in a more protracted time perspective of quality of recovery. Studies performed should also be procedure specific.

## Abbreviations

TAP block, transversus abdominal plane block.
